# Pattern-Dependent Mammalian Cell (Vero) Morphology on Tantalum/Silicon Oxide 3D Nanocomposites

**DOI:** 10.3390/ma11081306

**Published:** 2018-07-28

**Authors:** Hassan I. Moussa, Megan Logan, Wing Y. Chan, Kingsley Wong, Zheng Rao, Marc G. Aucoin, Ting Y. Tsui

**Affiliations:** 1 Department of Chemical Engineering, University of Waterloo, 200 University Avenue West, Waterloo, ON N2L 3G1 Canada; h2moussa@uwaterloo.ca (H.I.M.); m3logan@uwaterloo.ca (M.L.); w33chan@edu.uwaterloo.ca (W.Y.C.); kingsley.wong@edu.uwaterloo.ca (K.W.); z2rao@edu.uwaterloo.ca (Z.R.); marc.aucoin@uwaterloo.ca (M.G.A.); 2 Waterloo Institute of Nanotechnology, University of Waterloo, 200 University Avenue West, Waterloo, ON N2L 3G1, Canada

**Keywords:** tantalum, mammalian cells, morphology, adhesion, pseudopodia, nanoscale

## Abstract

The primary goal of this work was to investigate the resulting morphology of a mammalian cell deposited on three-dimensional nanocomposites constructed of tantalum and silicon oxide. Vero cells were used as a model. The nanocomposite materials contained comb structures with equal-width trenches and lines. High-resolution scanning electron microscopy and fluorescence microscopy were used to image the alignment and elongation of cells. Cells were sensitive to the trench widths, and their observed behavior could be separated into three different regimes corresponding to different spreading mechanism. Cells on fine structures (trench widths of 0.21 to 0.5 μm) formed bridges across trench openings. On larger trenches (from 1 to 10 μm), cells formed a conformal layer matching the surface topographical features. When the trenches were larger than 10 μm, the majority of cells spread like those on blanket tantalum films; however, a significant proportion adhered to the trench sidewalls or bottom corner junctions. Pseudopodia extending from the bulk of the cell were readily observed in this work and a minimum effective diameter of ~50 nm was determined for stable adhesion to a tantalum surface. This sized structure is consistent with the ability of pseudopodia to accommodate ~4–6 integrin molecules.

## 1. Introduction

Recent studies have used micro- and nanometer-scale engineered structures to mimic extracellular matrices and other biological structures, which have led to a groundbreaking understanding of the physical cues and molecular signal transduction pathways for integrin activated focal adhesion, protein adsorption, and pseudopodia formation [1,2,3,4,5,6,7,8,9]. Results have shown that cellular responses often depend on the mechanical properties, pattern structures, and surface chemistry of the microenvironment surrounding the cells [1,3,4,5,7,8,10,11,12,13,14,15,16,17,18,19,20]; however, work done thus far has been conducted with structures composed of monolithic materials having uniform surface chemical composition. Studies of cell behavior on nanocomposite surfaces are limited [1,3,17,18,19,20]. Recently, Baranes et al. [19] developed a novel technique that decorates polymeric nanofiber scaffolds with gold nanoparticles. This nanocomposite can promote longer outgrowth of neurites and control axonal elongation. Nanocomposites consisting of titania nanotubes/silver nanoparticles were successfully fabricated by Radtke et al. [20]. This material not only allows suitable adhesion of L929 murine fibroblast cells but also counteracts bacterial biofilm formation. While direct printing of protein [17,18] or functionalized nanoparticles [1,3] allow the study of cell behavior on patterned surfaces, the fabrication of these devices is restricted to 2D surfaces and cannot be extended to generate nanometer scale metal features. As a result, there is a knowledge gap when it comes to understanding cell behavior on 3D nanocomposite structures. Understanding this behavior is critical because of increasing interests in developing advanced medical implants made of different classes of materials. Results have already shown that biocomposites improve the bioactivity of the matrix materials [21,22,23], mechanical strength and stiffness [23], and antibacterial resistance [24,25]. Of materials that could be of interest as a biocomposite, tantalum stands out as being a bioactive metal [10] that is used in bulk, porous, and thin-film form in surgical implant devices [24,25,26,27,28,29,30,31].

The behavior of cells on these materials is in part characterized by their propensity to adhere to these surfaces, which is a complex process regulated by protein:protein interactions and cell signaling pathways. Of these protein:protein interactions, those that lead to the assembly of a focal adhesion are of interest because of their potential in probing the environment around 3D nanostructures. It is known that molecular binding between talin and the cytoplasmic tail of β1 integrin promotes integrin clustering leads to the assembly of a focal adhesion [32]. Given that each integrin molecule has a diameter of 8–12 nm [2,3] and based on observations of cells on c(-RGDfK-)-thiol-peptides-functionalized gold nanoparticle patterned adhesive surfaces, it has been suggested that a cluster of at least 6 ± 1 integrin molecules is required to produce robust focal adhesion [1]. Furthermore, Huang et al. [3] have suggested that a distance of ~70 nm between each integrin is required to sustain focal adhesion, which allows F-actin cytoskeleton formation by α-actinin in crosslinking.

Formation of focal adhesions and adherence to a surface is also driven by the surface itself. In recent work, Moussa et al. [12] examined mammalian cell adhesion on 2D tungsten/silicon oxide comb structure nanocomposites. In their work, surface topology was negligible given the precision-manufacturing of the chips used in the study. They showed that cells changed their shape to maximize the contact area with tungsten on the composite surfaces. Cells elongated on tungsten lines and aligned with the line axes. They were even able to develop a mathematical model based on this behavior to successfully predict cell morphology as a function of the composite pattern geometries. It is unclear if the same cell selective adhesion behaviors and the mathematical model developed hold for cells on tantalum–silicon oxide 3D nanocomposite surfaces.

The objective of this work was to investigate how cells reacted to being deposited on a 3D nanopatterned surface consisting of tantalum/silicon oxide parallel line/trench features ranging from ~210 nm to 100 μm. The driving hypothesis was that cells deposited on 3D metal patterned surfaces would display predictable behavior that could be linked to preferential contact to tantalum (a bioactive metal [21,22]) over silicon oxide, as is the case for 2D metal patterned surfaces [12]. Furthermore, it was hypothesized that the 3D patterning would allow greater exploration of cell pseudopodia and focal adhesions that could not be done on 2D surfaces. In this work, cell orientation relative to the patterned line/trench axes was characterized using fluorescence confocal microscopy and high-resolution scanning electron microscopy (SEM) techniques. Three regimes of attachment based on patterned surfaces have been observed and a mathematical relationship explaining the morphology of the cells as a function of the surface topology is proposed. Furthermore, unique observations of cell pseudopodia and focal adhesion have been made that showcase the limits of cellular projections.

## 2. Materials and Methods

### 2.1. Test Structure Fabrication

**Figure 1:**
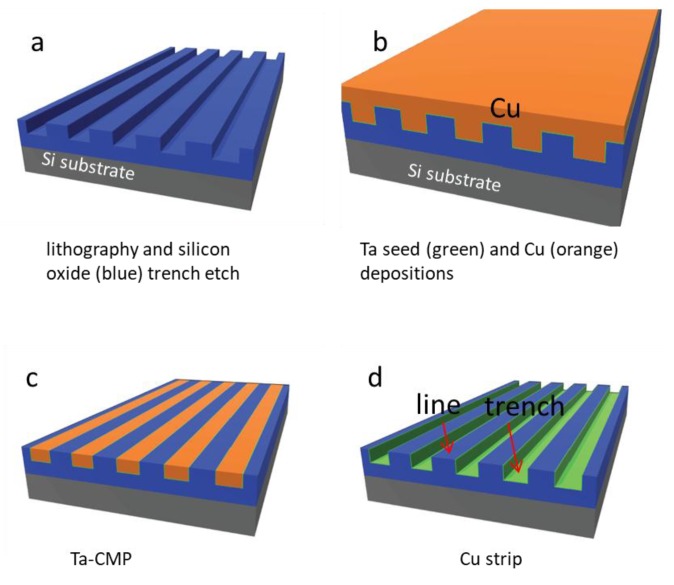
Schematic illustration of processing flow for tantalum (Ta) polished specimens. Parallel line/trench patterns were transferred to the silicon oxide thin film (blue) by lithography and dry etch techniques shown in (**a**). This is followed with Ta and copper (Cu) film depositions on the patterned structures in (**b**). Excess copper was then removed by using the copper and then followed with barrier metal chemical-mechanical polishing (CMP) in (**c**). The final step in (**d**) is copper strip by nitric acid.

Nanocomposite surfaces were prepared from materials supplied by Versum Materials, LLC (Tempe, AZ, USA). The original materials were made using industrial-scale advanced integrated circuit fabrication techniques, which include copper and tantalum chemical-mechanical polishing (CMP) and damascene integration on 200 mm silicon substrates [33,34,35] (Figure 1). Briefly, parallel-line comb structure patterns were transferred to the silicon oxide-coated substrate using lithography and dry-etching methods [33,34,36]. A thin tantalum seed layer and copper were deposited on the patterned structures. CMP [37,38] was used to remove excess copper and tantalum. Polishing continued until the silicon oxide lines were exposed as shown in Figure 1c. In-house, additional copper was removed by chemical stripping with ~9.4 M diluted nitric acid ACS Plus (Fisherbrand^®^, Fisher Scientific International Inc., Pittsburgh, PA, USA) for ~45 min. The final rinse consisted of deionized water and anhydrous ethanol. The finished device consisted of a parallel-line comb structure with trench sidewalls and bottom surfaces coated with a thin layer of tantalum, while the top surfaces of the lines contained exposed silicon oxide (Figure 1d).

### 2.2. Cell Culture and Deposition

Vero cells were deposited on the nanocomposite structures fabricated for this work. Detailed cell culture procedures have been described elsewhere [12]. Briefly, Vero cells obtained from the American Type Culture Collection (ATCC, Manassas, Virginia, USA) were cultured in tissue-culture-treated 75 cm^2^ flasks (Corning Falcon, VA, USA). Cells were cultured in DMEM/F12 media (Corning, New York, NY, USA), supplemented with 4 mM L-glutamine (Sigma-Aldrich, St. Louis, MO, USA) and 10% (*v*/*v*) Gibco™ fetal bovine serum (FBS, Thermo Fisher Scientific, Waltham, MA, USA). Cells were incubated on the structures in 5% CO_2_ atmosphere at 37 °C. The nanocomposite structures were sterilized with ethanol (70%) for 30 s and rinsed with Dulbecco’s phosphate-buffered saline (D-PBS) solution prior to the deposition of cells. All nanocomposite structures were inoculated with ~0.5 × 10^5^ cells/mL and incubated in 6-well tissue culture plates (Nunc, Thermo Scientific, Denmark) at 37 °C between 0.5 and 24 h.

### 2.3. Cell fixation, Staining, and Microscopy

After the prescribed incubation time, the nanocomposite structures were rinsed with D-PBS, and the adherent cells were fixed with 4% methanol-free formaldehyde (Sigma-Aldrich, Oakville, Canada) for 1 h following the same staining process detailed in Reference [12]. Briefly, cells were permeabilized with 0.1% Triton-X 100 (Sigma-Aldrich, Oakville, Canada) solution for 5 min and treated with 1% (*w*/*w*) bovine serum albumin (BSA) (Sigma-Aldrich, Oakville, Canada) prior to adding stains. Deep red CytoPainter F-Actin stain (ab112127 Abcam, Abcam Inc, Cambridge, MA, USA) and 4′,6-diamidino-2-phenylindole (DAPI) (Life Technologies, Waltham, MA, USA) were used to visualize the F-actin microfilaments and DNA molecules, respectively. Cells were immersed in a final solution containing Prolong Gold antifade reagent (Life Technologies, USA) and stored at 4 °C. Fluorescence confocal microscopy was carried out at the University of Guelph using a Leica TCS SP5 confocal fluorescence microscope (Leica, Wetzlar, Germany). The nanocomposite structures were inspected with wavelengths in the ranges of 436–482 nm (for DAPI) and 650–700 nm (for CytoPainter F-Actin).

Specimens for electron microscopy were fixed in 4% methanol-free formaldehyde and then dehydrated by submerging the specimens in increasingly concentrated ethanol solutions (50%, 75%, 95%, and 100% (*v*/*v*)) for at least 10 min each. Excess ethanol was removed during the final drying step with high purity nitrogen. Specimens were stored in a dry nitrogen box. A Zeiss 1550 field-emission scanning electron microscope (Carl Zeiss AG, Oberkochen, Germany) was used for the inspection with the electron gun operating at 7kV. No conductive coating was deposited on the specimens prior to imaging.

### 2.4. Measurement Parameters for Cell Alignment and Elongation

**Figure 2:**
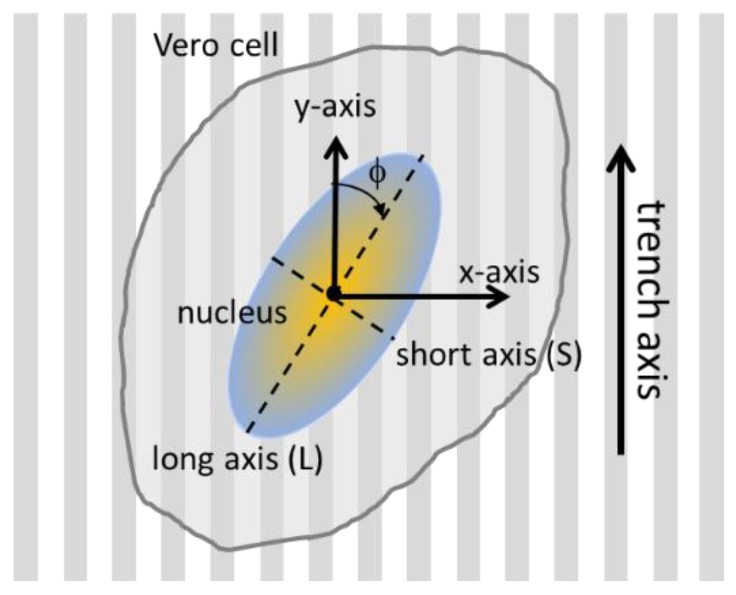
Schematic drawing of a cell adhered on parallel trench comb structure and their orientation parameters. The angle between the cell nucleus long axis and the trench axis is defined as (φ). The long and short axial lengths of the nucleus are L and W, respectively.

Several critical geometric parameters were characterized for each adherent cell. This included the nuclear orientation with respect to the trench axes, as well as their dimension along the long (L) and short (S) axes. A schematic illustrating these experimental parameters is shown in Figure 2. The angle between the long axis of an elliptical nucleus and the trench axis is defined as variable (φ). A nucleus that is oriented parallel to the trenches has an angle value of zero. The amount of cell elongation is characterized by the ratio between the two axial lengths (L/S). Cells that are severely elongated will have large L/S values. These cell characteristics were measured manually with Image Processing and Analysis in Java (ImageJ) software (National Institute of Mental Health, Bethesda, MD, USA). To avoid influences of the outer edges of the structured surface, only results 50 μm away from the periphery of the patterned area were included for the analyses.

### 2.5. Cell Contact Area on Tantalum and Silicon Oxide Surfaces

Contact area of cells with tantalum and silicon oxide surfaces was also characterized in this work. It is important to note that the area measured included the contributions from the trench sidewalls and bottom surfaces. The percent tantalum coverage area of an adherent cell, *A*(*Ta*), is defined as:

(1) $$A\left(Ta\right)=\overset{m}{\underset{k=1}{\sum }}\frac{A_{k}\left(Ta\right)}{A\left(total\right)}\times 100$$

where parameter (*m*) is the number of trenches contacting a cell. The contact area of cell with tantalum from each trench is *A_k_(Ta)*. *A(total)* is the total contact area between a cell and the comb structure surfaces.

## 3. Results and Discussions

### 3.1. Parallel Line Test Structures

As-fabricated parallel-line comb structures were inspected using SEM with specimens tilted at 70°. Typical micrographs of test structures with various line and trench widths are shown in Figure S1. Trenches were seen to be free of residue and the sidewalls were aligned nearly vertical with the trench bottom surfaces. The micrographs confirmed the trenches were ~500 nm deep. Nine different comb structures with trench widths of 0.21, 0.26, 0.5, 1, 2, 5, 10, 50, and 100 μm were fabricated as listed in Table 1. Except for the 0.21 and 0.26 μm trench structures, the rest of the comb structures had lines and trenches with equal widths. The line widths for the two smallest comb structures were 0.15 and 0.24 μm, respectively.

### 3.2. Effects of Patterning on Cell Alignment

**Figure 3:**
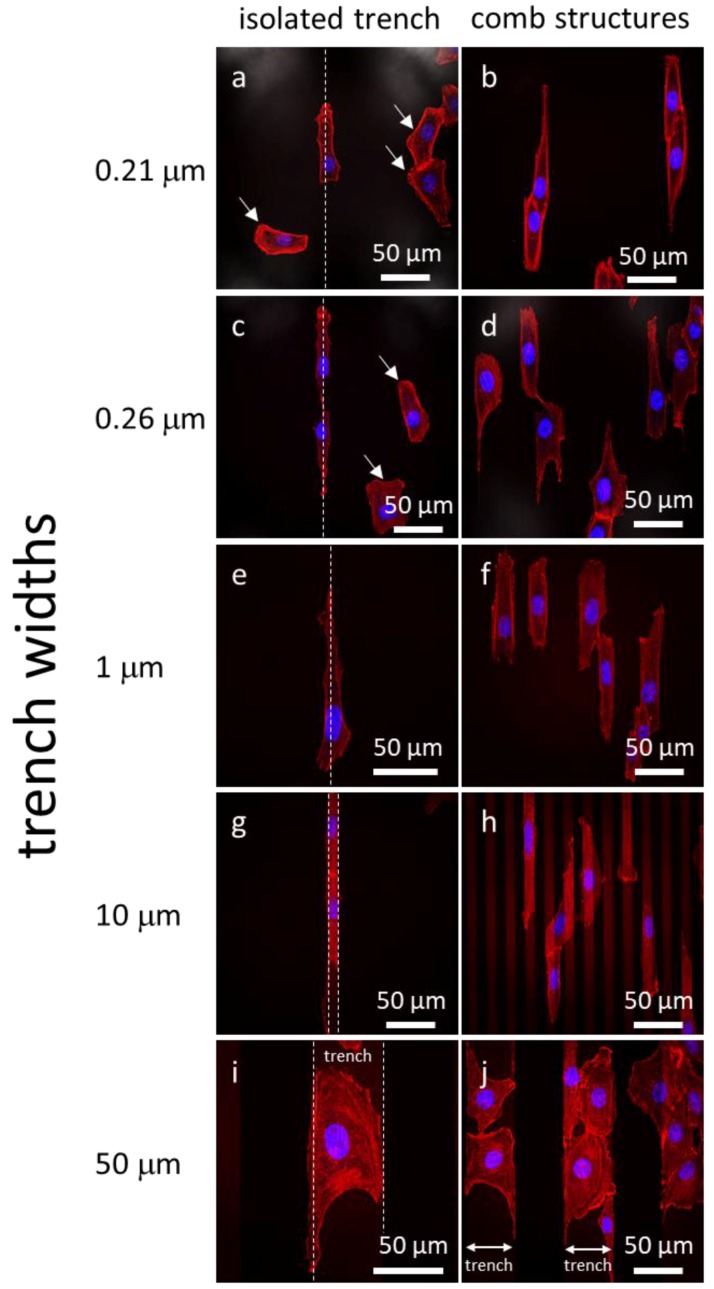
Fluorescence confocal micrographs of adherent cells on isolated trenches (a, c, e, g, i) and comb structures (b, d, f, h, j) with varying trench widths. White dash lines on the left column correspond to the isolated trench boundaries. The DNA is in blue (4′,6-diamidino-2-phenylindole (DAPI)) while F-actin microfilaments are in red (CytoPainter F-Actin). Cells adhered on the field silicon oxide are randomly oriented and labeled with arrows. They aligned with the trench axes when adhered on the patterned comb structures. Micrographs of 10 and 50 μm comb structures show cells prefer to adhere on the tantalum surface. Cells were incubated on the comb structures for 24 h.

**Figure 4:**
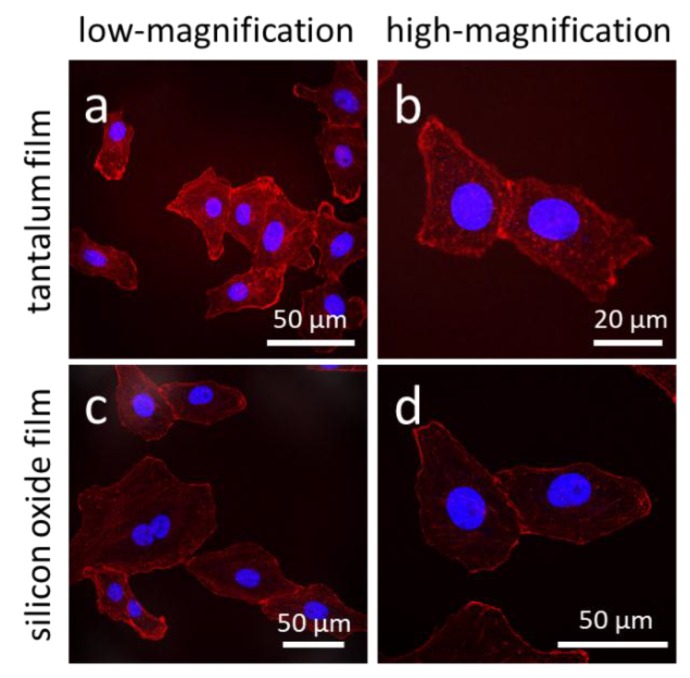
Fluorescence confocal micrographs of cells adhered on (**a,b**) blanket tantalum and (**c**,**d**) silicon oxide thin films. Images (**a**) and (**c**) were recorded at low-magnification while (**b**,**d**) at high-magnification. These cells are randomly oriented with irregular shapes. DNA was stained with DAPI in blue, while F-actin microfilaments were stained with CytoPainter F-Actin in red. Cells were incubated on the engineered surfaces for 24 h.

**Figure 5:**
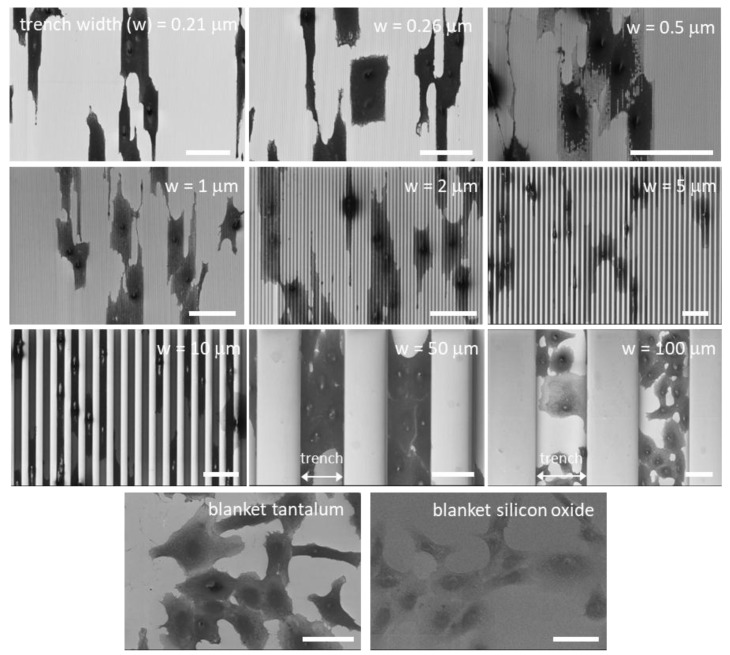
Typical top-down low-magnification scanning electron microscopy (SEM) micrographs of adherent cells on comb structures with various trench widths. Trench axes are in the vertical direction. Cells on blanket tantalum and silicon oxide films are also included. These micrographs show cells align with the trench axes on the comb structures. Cells preferentially adhered to tantalum surfaces on the 10, 50, and 100 μm comb structures. Scale bars correspond to 50 μm.

Representative fluorescence confocal micrographs of cells adhered to isolated trenches, and comb structures with varying trench widths, are shown in Figure 3. Five different trench width structures are presented in Figure 3: 0.21, 0.26, 1, 10, and 50 μm. Figure 3(a, c, e, g, i) shows micrographs of cells on isolated trenches. The trenches in these images are highlight by white dashed lines that show the boundaries of trenches. Figure 3(b, d, f, h, j) shows micrographs of cells on dense comb structures. DNA stained with DAPI can be seen in blue, while the F-actin microfilaments are those elements in red (stained with CytoPainter F-Actin). All of the cells were incubated on the comb structures for 24 h. Micrographs show cells on isolated trenches elongated and aligned with the trenches. Nearby cells on the blanket silicon oxide, highlighted with white arrows, were randomly oriented and had irregular shapes. On the dense comb structures, displayed in the Figure 3(b, d, f, h, j), cells and their nuclei aligned with the trench axes in the same manner as those on the isolated lines. In addition, the majority of cells on the 10 and 50 μm-wide comb structures localized in the trenches where the sidewalls and the bottom surfaces were coated with tantalum. These micrographs show that cells were more likely to reside in the trenches than on the silicon oxide surfaces. As a comparison, micrographs of cells on flat blanket tantalum and silicon oxide are displayed in Figure 4. Cells adhered and spread randomly on both the tantalum and silicon oxide blanket surfaces. The cells on the comb structures were also inspected using high-resolution SEM. Top-down low-magnification micrographs of adherent cells on the different surfaces are displayed in Figure 5. These cells were incubated for 24 h. Micrographs of the 10, 50, and 100 μm comb structure revealed that cells preferentially adhered to tantalum, consistent with the confocal images shown in Figure 3.

**Figure 6:**
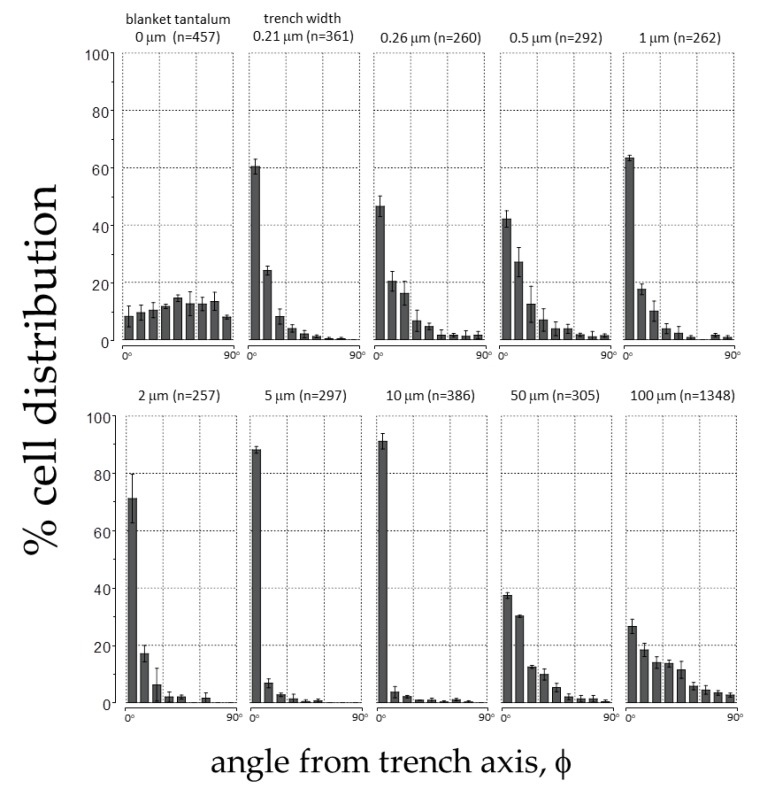
Distribution of adherent cell orientation (in percentage) on blanket tantalum film and comb structures with trench widths between 0.21 μm and 100 μm. The cell orientation is characterized as the angle (φ) between the nuclei long axis and the patterned trench axes. The number of adherent cells characterized (n) in each area is also presented in the plots. Each bin represents the percentage of cell population in a ±10° interval deviated from the trench axis. Results show that cells aligned preferentially with the trench axes.

To quantify this effect, the angular position of the cell’s nucleus with respect to the direction of the patterned lines (φ, see Figure 2) was measured after a 24 h incubation period. Distributions representing a population of cells on each surface were generated (Figure 6). Cells on the blanket tantalum were randomly oriented with no preferred orientation. In contrast, a significant number of cells aligned with the trench axes when they were incubated on the comb structures with trench width in the range of 0.21 and 10 μm. More than 91% of the nuclei were oriented within ±10° of the trench axes on the 10 μm comb structures. Cells became increasingly randomized when widths were wider than 50 μm.

**Figure 7:**
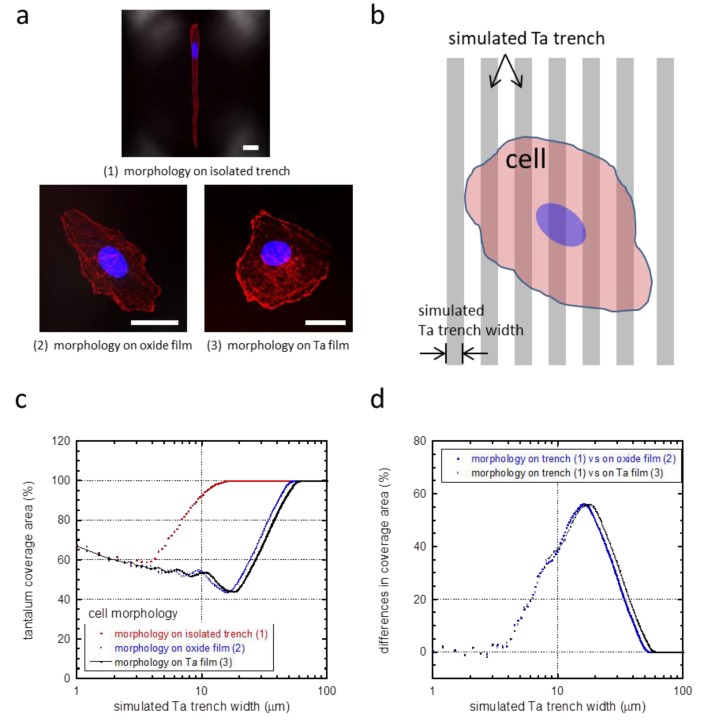
(**a**) Cell morphologies that were used to calculate the tantalum coverage: (**1**) on isolated trenches, (**2**) on silicon oxide films, and (**3**) on tantalum (Ta) films. Scale bars correspond to 25 μm. A schematic drawing illustrating a model cell on simulated parallel trenches and lines is shown in (**b**). (**c**) Percent tantalum coverage calculated from our mathematical simulation of cells with three different morphologies. Tantalum coverage converged at trench widths smaller than 2 μm and larger than 60 μm for all morphologies. (**d**) Difference in tantalum coverage between elongated cell morphology (**1**) and a random-shaped cell morphology on blanket oxide (**2**) and tantalum films (**3**).

A possible mechanism for this phenomenon is selective adhesion to tantalum compared to silicon oxide [12]. To investigate this, a mathematical equation (Equation (1)) was developed to investigate whether these cells changed their morphology and shape to maximize their contact with the tantalum surface, not unlike what has been previously shown for tungsten surfaces [12]. In this investigation, no preference was given as to whether the tantalum surface was part of the trench sidewall or bottom. Model cell morphologies observed on isolated trenches (Figure 7a(1)) or blanket surfaces (Figure 7a(2,3)) were used to evaluate surface coverage on different geometries, i.e., model cells were superimposed on simulated comb structures as shown in Figure 7b. For each model cell (Figure 7a(1–3)), simulated tantalum coverage is plotted as a function of the trench width (Figure 7c). The results showed that the coverage area of tantalum initially decreased for all cell morphologies with increasing trench width. This is due to a smaller area contribution from the trench sidewalls. Once a minimum was reached, the tantalum coverage increased until the cell was fully sitting atop the tantalum surface, i.e., the model cell was smaller than the trench width. We have previously suggested that cells will change their shape and orientation when coverage by the elongated cell (Figure 7a(1)) is larger than that of the randomly shaped cell (Figure 7a(2,3)). In Figure 7d, coverage difference increases for trench widths from 3 to ~60 μm. Hence, there is an increase tendency for cells to elongate in this range. There is no incentive for cell elongation when widths are larger than 60 μm—however, it is also expected that, beyond 60 μm, cells will not appear on the silicon oxide surface.

The experimental results shown in Figure 6 support, in part, the notion that the cells are trying to maximize their contact area; however, the gross morphology of the cells on comb structures having trench/line widths less than 3 μm is not as random as those observed on blanket tantalum or silicon oxide. Therefore, although the model does give us a means to predict the behavior of cells, the 3D nature of the surface adds some additional complexity that is not captured by the model. 

### 3.3. Regimes Created by the 3D Nature of the Nanocomposite Surfaces

**Figure 8:**
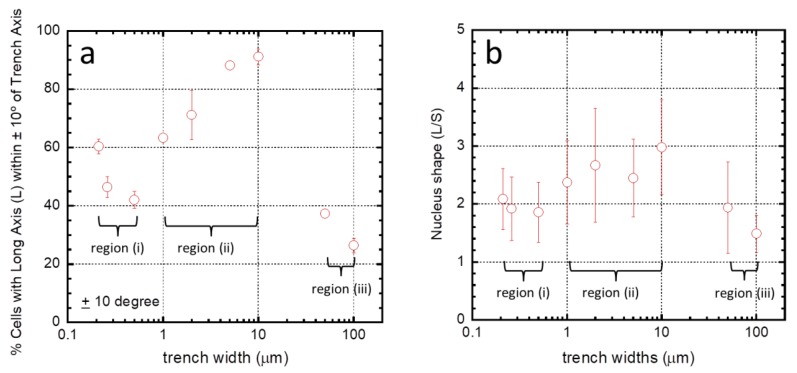
(**a**) Percentage of cells in which the long axis (L) of their nucleus aligned within ±10° of the trench axis when adhered on comb structures with varying trench widths. Results show cell orientation depends strongly on trench width. Three different regimes are observed with widths between (i) 0.21–0.5 μm, (ii) 1–10 μm, and (iii) 50–100 μm; (**b**) nucleus shape (as defined by the ratio short (S) and long axes (L)) as a function of trench widths. Three different regions can be identified based on the relationships between the length ratios and width of trenches—between (i) 0.21–0.5 μm; (ii) 1–10 μm; and (iii) 50–100 μm.

To help visualize the influences of comb structure geometries, the proportion of the population of cells aligned within ±10° from the axes was plotted as a function of trench widths (Figure 8a). Three regimes emerged based on trench widths: (i) for widths between 0.21–0.5 μm; (ii) between 1–10 μm; and (iii) for 50–100 μm. Alignment of cells on the fine comb structures in region (i) rapidly decreased with increasing trench width. In contrast, cells in region (ii) showed an opposite behavior with an increasing preference for alignment as trench widths increased. In region (iii), the number of cells aligned with the axes drops abruptly—presumably heading towards a random distribution as seen with the blanket tantalum surface. The shape of the nucleus characterized by the ratio of the long and short axes (L/S, see Figure 2) was also influenced by the surface structure (Figure 8b). Similar regions for the shape of the nucleus can be defined as was done for the angular orientation of the nucleus (Figure 8a).

#### Nanometer Scale Morphology of Cells in Regions (i)–(iii)

**Figure 9:**
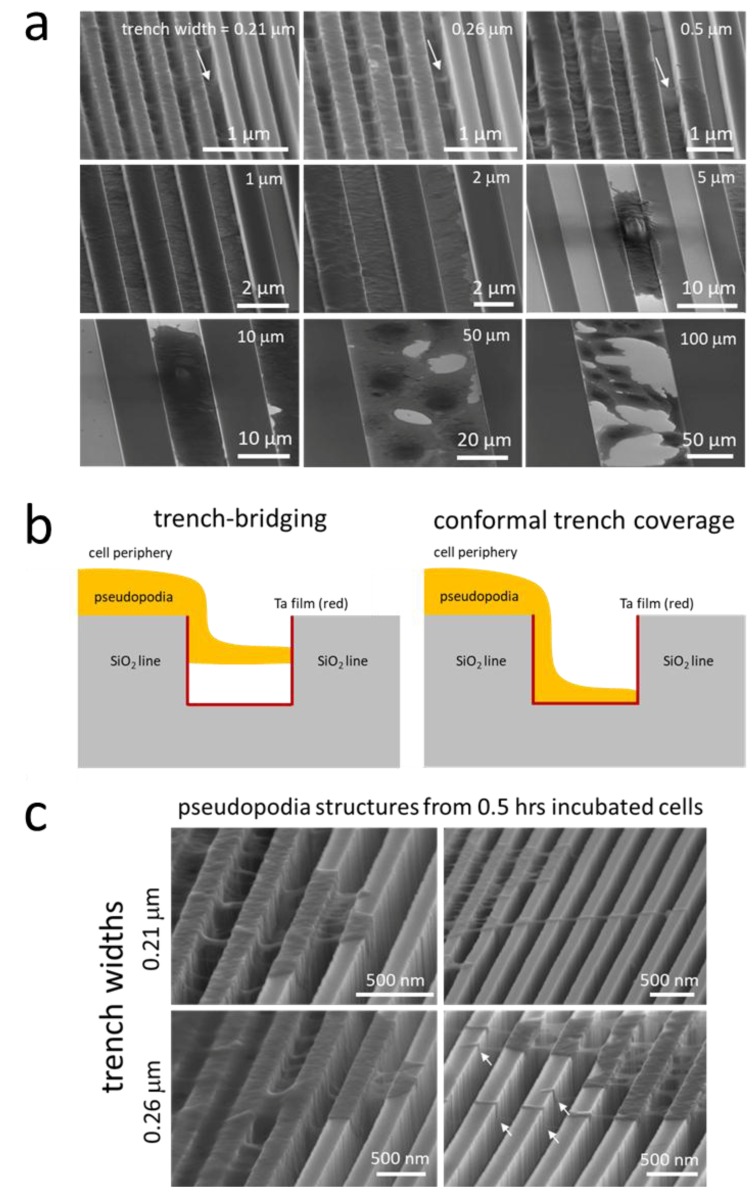
(**a**) Representative 70°-tilted high-magnification SEM micrographs of adherent cells on comb structures with various trench widths. White arrows indicate examples of pseudopodia that bridged across the trenches; (**b**) schematic illustrations of two spreading behaviors of pseudopodia across trenches—trench bridging and conformal trench coverage; (**c**) typical 70°-tilted SEM micrographs of cells incubated on parallel trench structures with trench widths of 0.21 and 0.26 μm for 0.5 h. White arrows indicate examples of pseudopodia that bridged across the trenches.

Further probing of the cells in these three distinct regions was done using high-resolution SEM at a high-tilt angle (70°). The upper three panels of Figure 9a show that cell pseudopodia formed bridges across the trenches as highlighted with arrows in the image. These bridges do not appear to make contact with the trench bottom and appear suspended above the surface. The anchor points of these bridges were often located more than ~100 nm deep into the trenches. In some instances, such as those highlighted for trench widths of 0.21 and 0.26 μm, the pseudopodia did not migrate up to the top of the trenches after adhering to the sidewalls. A schematic drawing illustrating this trench-bridging behavior is shown in Figure 9b. While trench bridging was characteristic of the 0.21 and 0.26 μm comb structures, only two pseudopodia bridges were observed for the 0.5 μm trenches. In fact, the pseudopodia adhered as a conformal covering of the surface for surfaces with trench widths of 0.5 μm.

The trench-bridging morphology appears to begin shortly after the seed-plating process. High-magnification micrographs of cells on 0.21 and 0.26 μm comb structures after 0.5 h of incubation are shown in Figure 9c. The images clearly show pseudopodia near the cell periphery bridged the trench and adhered to the adjacent sidewalls, similar to those incubated for 24 h (see Figure 9a). Furthermore, it can be seen from these images that some of the anchor points for these bridges were located on the sidewalls deep inside the trenches. Despite the ability of the pseudopodia to enter the trenches, there must be some type of physical limitation that will not allow the majority of the cell to recede into the trenches. Additional micrographs of cells on 0.21 and 0.26 μm comb structures are shown in Supplemental Figures S2–S6 to demonstrate the reproducibility of the bridge formation. Examples of long filaments connecting neighboring cells are shown in Figure S7.

**Figure 10:**
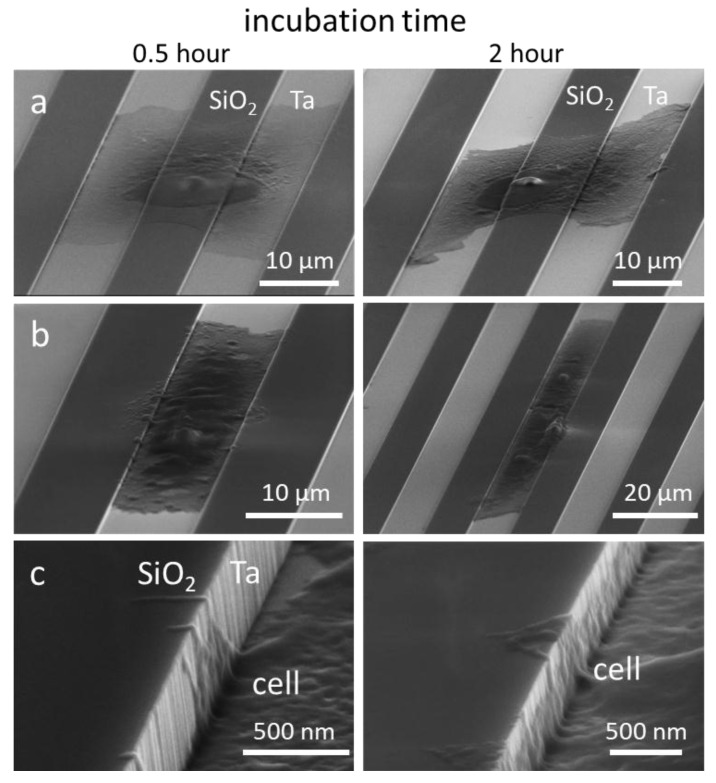
Typical 70°-tilted SEM micrographs of 0.5 and 2 h incubated cells on 10 μm comb structures with cell nuclei centered on (**a**) silicon oxide lines and (**b**) tantalum coated trenches. (**c**) High-magnification of pseudopodia on the trench sidewalls.

In region (ii), where the trench widths are between 1 and 10 μm, conformal surface coverage was the dominant pseudopodia-spreading characteristic (see Figure 9a). This morphology was also observed during the early stages of attachment on the 10 μm comb structures (Figure 10). The left- and right-column micrographs are of cells incubated for 0.5 and 2 h, respectively. Figure 10a,b shows cells adhered and spread on both the silicon oxide lines and the tantalum-coated trenches; however, the cells preferentially spread along the tantalum trenches. High-magnification micrographs of the 10 μm trench sidewalls showed cytoplasm filaments with diameters smaller than 100 nm extending in a direction perpendicular to the trench axes (Figure 10c), moving up the sidewalls and appearing to probe the surrounding microenvironments. Although this demonstrates that cells have the capacity to overcome the sidewall barriers, trench widths are wide enough for cells to preferentially move in the direction of the trench axes.

**Figure 11:**
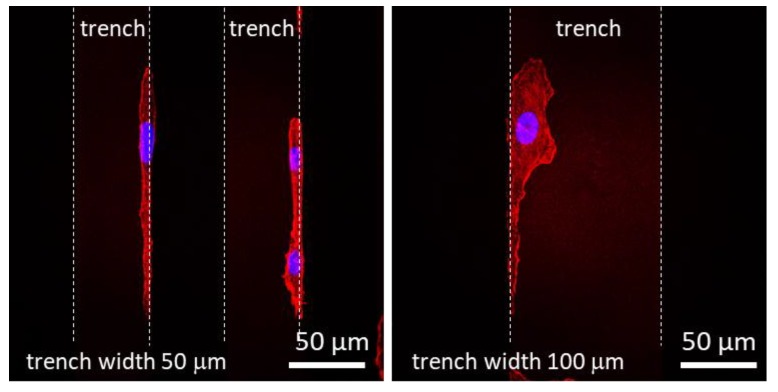
Fluorescence confocal micrographs of adherent cells elongated on the 50 and 100 μm wide trenches at the trench sidewall and bottom junctions. Dash lines correspond to the trench boundaries.

In region (iii), the trenches are wide enough to accommodate multiple cells as shown in Figure 3, Figure 4 and Figure 5. The morphology of the majority of the cells in these trenches approached that of cells on blanket tantalum and silicon oxide. However, a small portion of cells exhibited a unique morphology (Figure 11), where the cells seemed to favor the 90° corner between the trench sidewall and the bottom surface. In any case, when the trench widths are large enough to contain the entire cell, the cells localize on the tantalum surface preferentially over the silicon oxide surface.

### 3.4. Critical Focal Adhesion Dimensions

Figure 9a,c shows the smallest bridge anchor points for short and long-stranded pseudopodia on the sidewalls have diameters as small as ~50 nm. In theory, such a size could accommodate a cluster of 4 to 6 integrin molecules (diameter of 8–12 nm [2]). Our results appear to agree with Arnold et al. [1] and Coussen et al. [2], who suggested that at least three to six integrins are required for paxillin accumulation and actin bundle formation, which leads to stable focal adhesions on 2D surfaces. This is believed to be the first direct measurement of the smallest possible mammalian cell pseudopodia that can produce stable focal adhesion on a 3D structure. Furthermore, the cytoplasmic projections seem to be able to jut out of the cell without needing a surface on which to spread. Unlike previous cell adhesion studies [1,2,3], where surfaces were pretreated with known integrin ligands, such as c(-RGDfK-)-thiol peptides, arginine-glycine-aspartic acid (RGD), or FN7-10 fibronectin, our nanocomposite materials were not coated with any ligands prior to cell deposition. It is likely that sidewall adhesion was facilitated by components in FBS in the cell culture medium. Moussa et al. [12] and Teixeira et al. [39] have shown that when cells were incubated on parallel comb structures in the absence of FBS, alignment of cells reduced dramatically. Regardless of the possible ligand, ligand adsorption and integrin activation processes happened within 0.5 h after cell deposition (Figure 9 and Figure 10).

## 4. Conclusions

Cell adhesion and the resulting morphologies of Vero cells on a tantalum/silicon oxide nanocomposite were characterized. High-resolution SEM micrographs revealed nanometer-scale pseudopodia that formed bridges between trench sidewalls when surfaces had trench widths smaller than 0.5 μm. Cytoplasmic projections formed conformal coats on wider trench structures and did not show bridge formation. Three different cell alignment regimes were identified based on cell nuclei orientation dependence with trench widths. A decrease of the cell nuclei alignment performance in region (i) with trench widths from 0.21 to 0.5 μm is attributed to the reduced physical barrier for cells to spread in direction perpendicular to the trench axes. The increase of alignment performance for trench width in the range of 1 and 10 μm (region (ii)) is believed to be driven by the cell-selective material adhesion behaviors on tantalum-coated trenches. This mechanism also explains the increase of random cell orientation and shape for trench widths larger than 50 μm (region (iii)). One of the potential applications of these nanocomposites is in the preparation of the exterior surface of surgical implants to allow enhanced osseointegration by controlling cell alignment.

## Figures and Tables

**Table 1:** A data summary of test structure dimensions, number of cells inspected (n), percent population of cells with 10° > φ > −10° of the line axis, and axis length ratio (L/S). The culture media initial cell concentration used was ~ 0.5 × 10^5^ cells/mL and cells were incubated on the comb structures for 24 h. Data spreads correspond to one standard deviation.

Structure	Line/Trench Widths (μm)	Inspected Comb Structure Area (mm^2^)	Number of Cells Sampled (n)	L/S	% of Population Aligned ± 10° from Trenches (10° > φ > −10°)
1	0.15/0.21	1.8	361	2.09 ± 0.53	60.4 ± 2.6
2	0.24/0.26	1.8	260	1.92 ± 0.55	46.5 ± 3.6
3	0.5/0.5	1.8	292	1.86 ± 0.52	42.1 ± 2.9
4	1/1	1.8	262	2.38 ± 0.72	63.4 ± 0.9
5	2/2	1.8	257	2.67 ± 0.98	71.2 ± 8.5
6	5/5	1.8	297	2.45 ± 0.67	88.2 ± 1.2
7	10/10	1.8	386	2.98 ± 0.82	91.2 ± 2.7
8	50/50	1.8	305	1.94 ± 0.79	37.4 ± 1.0
9	100/100	6.6	1348	1.49 ± 0.32	26.6 ± 2.5
10	blanket Ta film	1.8	457	1.64 ± 0.44	8.1 ± 3.6

## Supplementary Materials

The following are available online at http://www.mdpi.com/1996-1944/11/8/1306/s1. Figure S1: SEM micrograph of cross-sectioned specimens, Figure S2: Cell on 0.21 μm comb structure incubated for 0.5 hours (1), Figure S3: Cell on 0.21 μm comb structure incubated for 0.5 hours (2), Figure S4: Cell on 0.21 μm comb structure incubated for 0.5 hours (3), Figure S5: Cell on 0.26 μm comb structure incubated for 0.5 hours (1), Figure S6: Cell on 0.26 μm comb structure incubated for 0.5 hours (2), Figure S7: Long single-stranded filopodia on 0.21 μm comb structure.

## References

[1] Arnold M., Schwieder M., Blümmel J., Cavalcanti-Adam E.A., López-Garcia M., Kessler H., Geiger B., Spatz J.P. (2009). Cell interactions with hierarchically structured nano-patterned adhesive surfaces. Soft Matter.

[2] Coussen F., Choquet D., Sheetz M.P., Erickson H.P. (2002). Trimers of the fibronectin cell adhesion domain localize to actin filament bundles and undergo rearward translocation. J. Cell Sci..

[3] Huang J., Gräter S.V., Corbellini F., Rinck S., Bock E., Kemkemer R., Kessler H., Ding J., Spatz J.P. (2009). Impact of order and disorder in RGD nanopatterns on cell adhesion. Nano Lett..

[4] Ridley A.J., Hall A. (1992). The small GTP-binding protein rho regulates the assembly of focal adhesions and stress fibres in response to growth factors. Cell.

[5] Hadjiantoniou S.V., Sean D., Ignacio M., Godin M., Slater G.W., Pelling A.E. (2016). Physical confinement signals regulate the organization of stem cells in three dimensions. J. R. Soc. Interface.

[6] Kang H., Wong D.S.H., Yan X., Jung H.J., Kim S., Lin S., Wei K., Li G., Dravid V.P., Bian L. (2017). Remote Control of Multimodal Nanoscale Ligand Oscillations Regulates Stem Cell Adhesion and Differentiation. ACS Nano.

[7] Liang E.I., Mah E.J., Yee A.F., Digman M.A. (2017). Correlation of focal adhesion assembly and disassembly with cell migration on nanotopography. Integr. Biol..

[8] Lee S., Kim D., Kim S.-M., Kim J.-A., Kim T., Kim D.-Y., Yoon M.-H. (2015). Polyelectrolyte multilayer-assisted fabrication of non-periodic silicon nanocolumn substrates for cellular interface applications. Nanoscale.

[9] Mcguire A.F., Santoro F., Cui B. (2018). Interfacing Cells with Vertical Nanoscale Devices: Applications and Characterization. Annu. Rev. Anal. Chem..

[10] Huo W.T., Zhao L.Z., Yu S., Yu Z.T., Zhang P.X., Zhang Y.S. (2017). Significantly enhanced osteoblast response to nano-grained pure tantalum. Sci. Rep..

[11] Kong H.J., Hsiong S., Mooney D.J. (2007). Nanoscale cell adhesion ligand presentation regulates nonviral gene delivery and expression. Nano Lett..

[12] Moussa H.I., Logan M., Siow G.C., Phann D.L., Rao Z., Aucoin M.G., Tsui T.Y. (2017). Manipulating mammalian cell morphologies using chemical-mechanical polished integrated circuit chips. Sci. Technol. Adv. Mater..

[13] Nobes C.D., Hall A. (1995). Rho, Rac, and Cdc42 GTPases regulate the assembly of multimolecular focal complexes associated with actin stress fibers, lamellipodia, and filopodia. Cell.

[14] Persson H., Li Z., Tegenfeldt J.O., Oredsson S., Prinz C.N. (2015). From immobilized cells to motile cells on a bed-of-nails: Effects of vertical nanowire array density on cell behaviour. Sci. Rep..

[15] Wood A. (1988). Contact guidance on microfabricated substrata: The response of teleost fin mesenchyme cells to repeating topographical patterns. J. Cell Sci..

[16] Xie X., Xu A.M., Angle M.R., Tayebi N., Verma P., Melosh N.A. (2013). Mechanical model of vertical nanowire cell penetration. Nano Lett..

[17] Poudel I., Lee J.S., Tan L., Lim J.Y. (2013). Micropatterning–retinoic acid co-control of neuronal cell morphology and neurite outgrowth. Acta Biomater..

[18] Tay C.Y., Yu H., Pal M., Shing W., Soon N., Woei K., Tai D., Poh L. (2010). Micropatterned matrix directs differentiation of human mesenchymal stem cells towards myocardial lineage. Exp. Cell Res..

[19] Baranes K., Shevach M., Shefi O., Dvir T. (2016). Gold Nanoparticle-Decorated Scaffolds Promote Neuronal Differentiation and Maturation. Nano Lett..

[20] Radtke A., Jędrzejewski T., Kozak W., Sadowska B., Więckowska-Szakiel M., Talik E., Mäkelä M., Leskelä M., Piszczek P. (2017). Optimization of the Silver Nanoparticles PEALD Process on the Surface of 1-D Titania Coatings. Nanomaterials.

[21] Balla V.K., Bodhak S., Bose S., Bandyopadhyay A. (2011). Porous Tantalum Structures for Bone Implants: Fabrication, Mechanical and In vitro Biological Properties. Acta Biomater..

[22] Balla V.K., Bose S., Davies N.M., Bandyopadhyay A. (2010). Tantalum—A Bioactive Metal for Implants. JOM.

[23] Liu H., Webster T.J. (2010). Mechanical properties of dispersed ceramic nanoparticles in polymer composites for orthopedic applications. Int. J. Nanomed..

[24] Kazemzadeh-Narbat M., Kindrachuk J., Duan K., Jenssen H., Hancock R.E.W., Wang R. (2010). Antimicrobial peptides on calcium phosphate-coated titanium for the prevention of implant-associated infections. Biomaterials.

[25] Pezzotti G., Marin E., Adachi T., Lerussi F., Rondinella A., Boschetto F., Zhu W., Kitajima T., Inada K., McEntire B.J. (2018). Integrating the Biologically Friendly Chemistry of Si_3_N_4_ Bioceramics to Produce Antibacterial, Osteoconductive, and Radiolucent PEEK Spinal Implants. Macromol. Biosci..

[26] Balla V.K., Banerjee S., Bose S., Bandyopadhyay A. (2010). Direct laser processing of a tantalum coating on titanium for bone replacement structures. Acta Biomater..

[27] Black J. (1994). Biological Performance of Tantalum. Clin. Mater..

[28] Levine B.R., Sporer S., Poggie R.A., della Valle C.J., Jacobs J.J. (2006). Experimental and clinical performance of porous tantalum in orthopedic surgery. Biomaterials.

[29] Matassi F., Botti A., Sirleo L., Carulli C., Innocenti M. (2013). Porous metal for orthopedics implants. Clin. Cases Miner. Bone Metab..

[30] Tang Z., Xie Y., Yang F., Huang Y., Wang C., Dai K., Zheng X., Zhang X. (2013). Porous Tantalum Coatings Prepared by Vacuum Plasma Spraying Enhance BMSCs Osteogenic Differentiation and Bone Regeneration In Vitro and In Vivo. PLoS ONE..

[31] Ren B., Zhai Z., Guo K., Liu Y., Hou W., Zhu Q., Zhu J. (2015). The application of porous tantalum cylinder to the repair of comminuted bone defects: A study of rabbit firearm injuries. Int. J. Clin. Exp. Med..

[32] Wozniak M.A., Modzelewska K., Kwong L., Keely P.J. (2004). Focal adhesion regulation of cell behavior. Biochim. Biophys. Acta Mol. Cell Res..

[33] Doering R., Nishi Y. (2007). Handbook of Semiconductor Manufacturing Technology.

[34] Chen W.-K. (2007). The VLSI Handbook.

[35] Li Y. (2007). Microelectronic Applications of Chemical Mechanical Planarization.

[36] Van Zant P. (2014). Microchip Fabrication: A Practical Guide to Semiconductor Processing, Sixth ed..

[37] Shi X., Murella K., Schlueter J.A., Choo J.O. (2015). Chemical Mechanical Polishing Slurry Compositions and Method Using the Same for Copper and through-Silicon via Applications. U.S. Patent.

[38] Shi X., Palmer B.J., Sawayda R.A., Coder F.A., Perez V. (2013). Method and Composition for Chemical Mechanical Planarization of a Metal. U.S. Patent.

[39] Teixeira A.I., Abrams G.A., Bertics P.J., Murphy C.J., Nealey P.F. (2003). Epithelial contact guidance on well-defined micro- and nanostructured substrates. J. Cell Sci..

