# Supplementary material for: Pattern-Dependent Mammalian Cell (Vero) Morphology on Tantalum/Silicon Oxide 3D Nanocomposites
Source: Materials (Basel). 2018 Jul 28;11(8):1306. doi: 10.3390/ma11081306 (PMC6117680; doi:10.3390/ma11081306)
Supplement: Supplementary file 1 [file materials-11-01306-s001.zip › materials-328571-SI.pdf]

Supplement figures

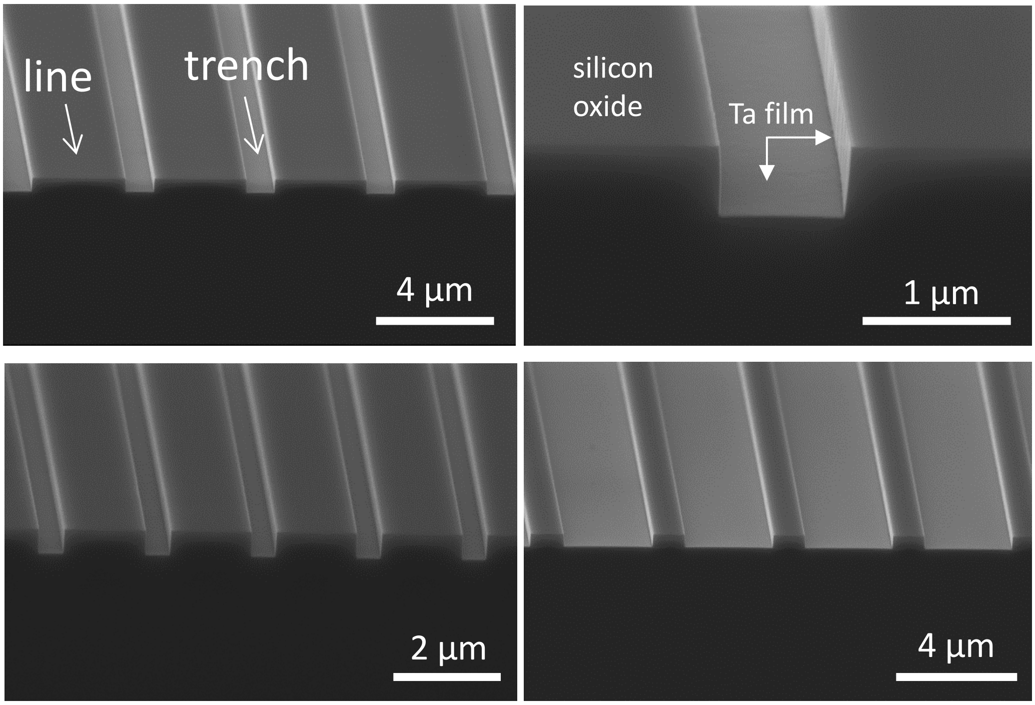

Figure S1. Typical 70° tilted SEM micrographs of cross-sectioned specimen with various line and trench widths. Sidewalls and trench bottoms are covered with tantalum film while the line top surface have silicon oxide exposed.

0.21 um trench, 0.5 hours of incubation

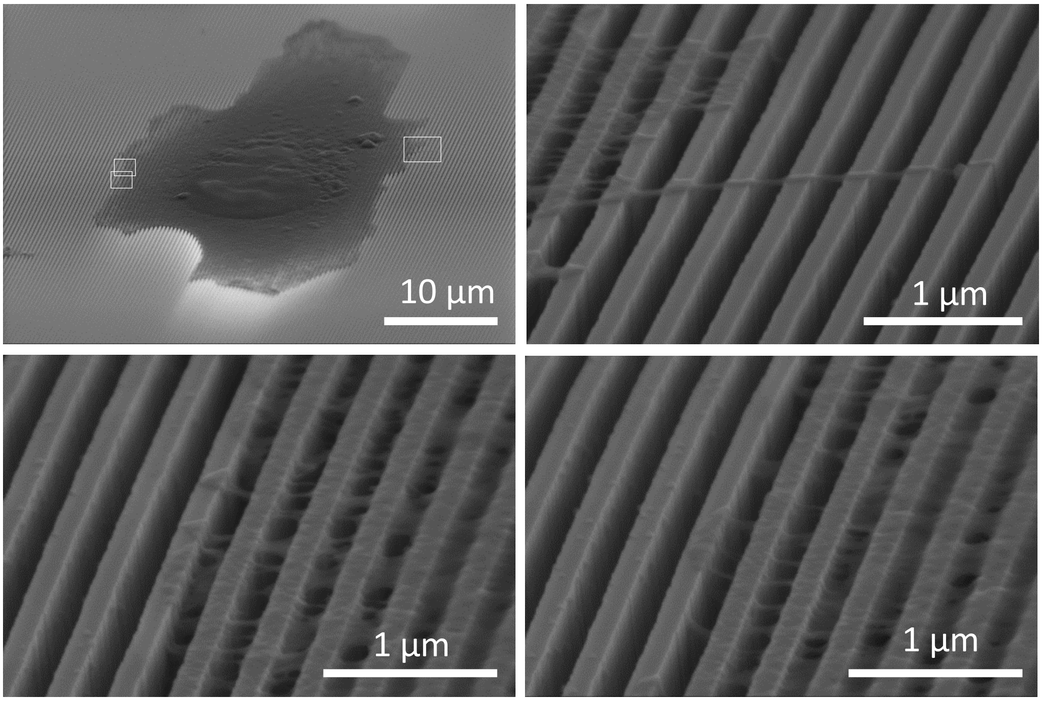

Figure S2 . Representative 70° tilted SEM micrograph of a cell incubated for 0.5 hour on a 0.21 µm trench comb structure.

0.21 um trench, 0.5 hours of incubation

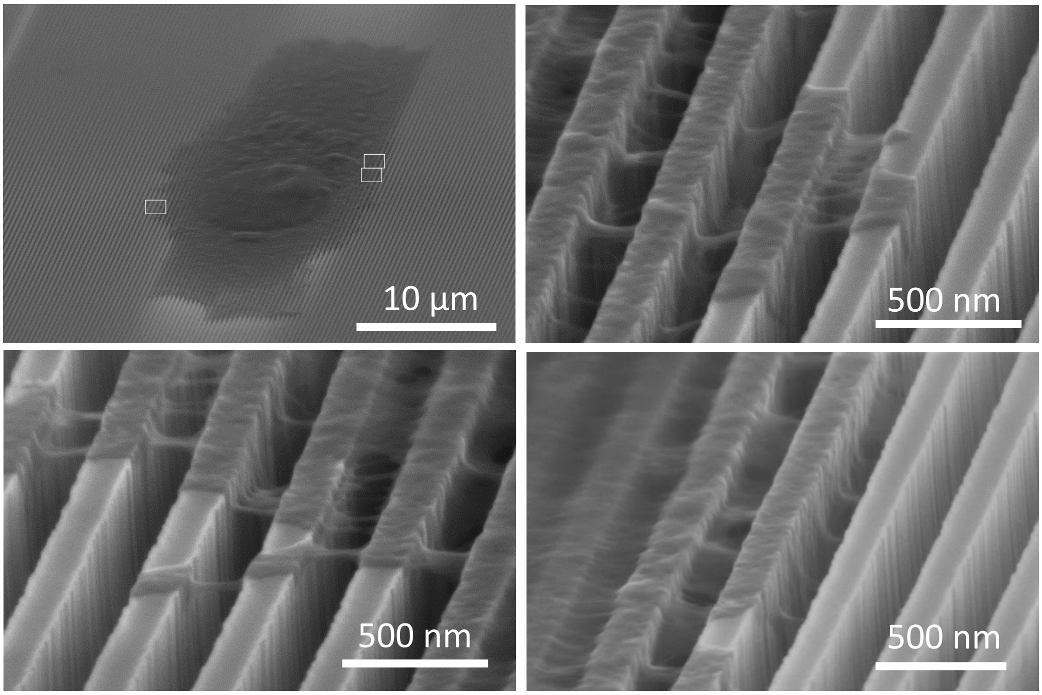

Figure S3. Representative 70° tilted SEM micrograph of a cell incubated for 0.5 hour on a 0.21 μm comb structure.

0.21 um trenches, 0.5 hours incubation

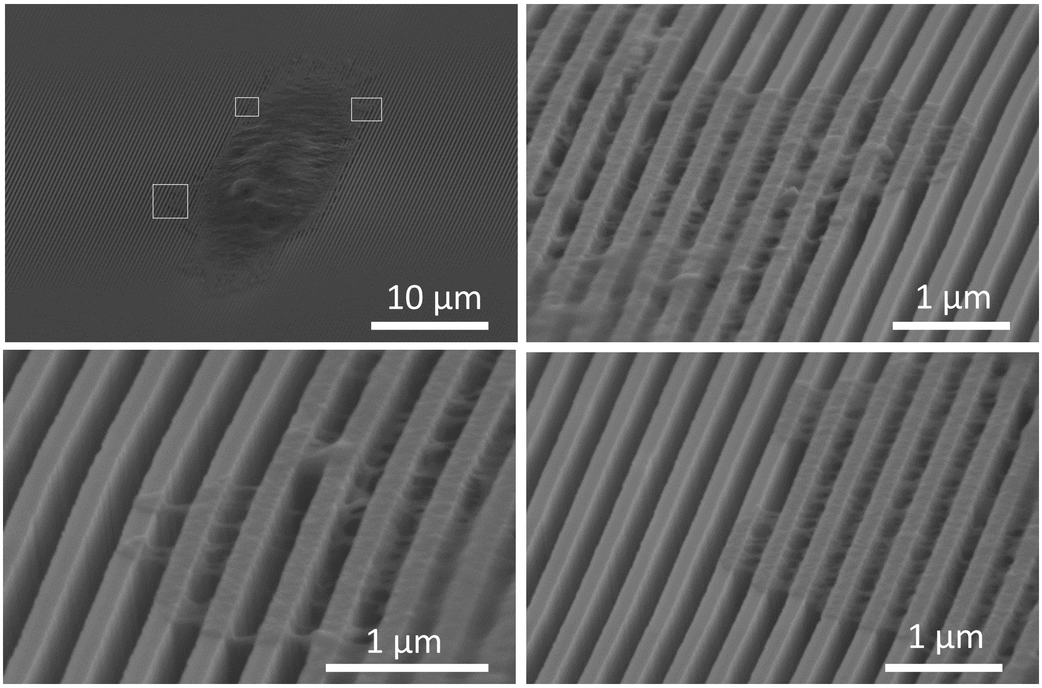

Figure S4. Representative 70° tilted SEM micrograph of a cell incubated for 0.5 hour on a 0.21 μm comb structure.

0.26 um trenches, 0.5 hours incubation

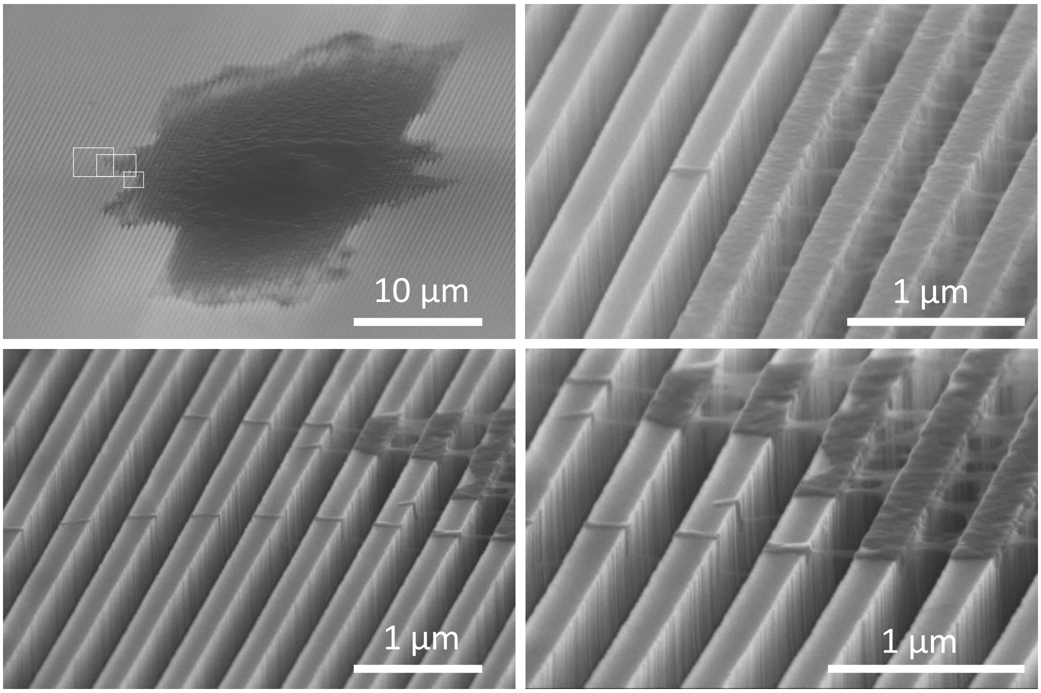

Figure S5. Representative 70° tilted SEM micrograph of a cell incubated for 0.5 hour on a 0.26 µm comb structure.

0.26 um trenches, 0.5 hours incubation

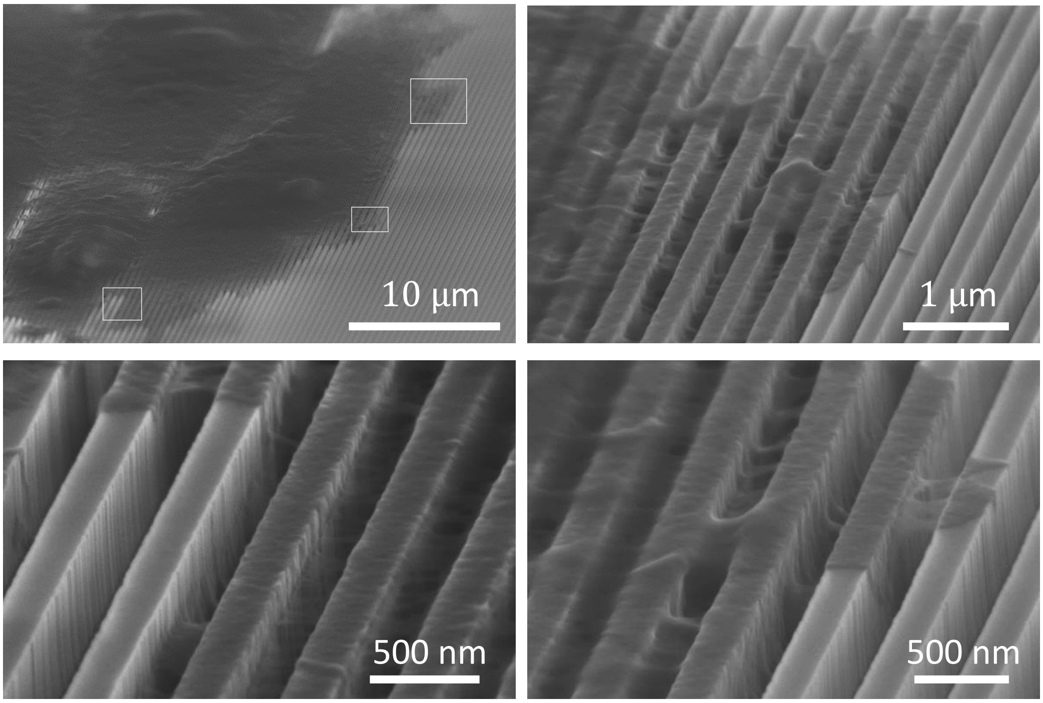

Figure S6. Representative 70° tilted SEM micrograph of a cell incubated for 0.5 hour on a 0.26 μm comb structure.

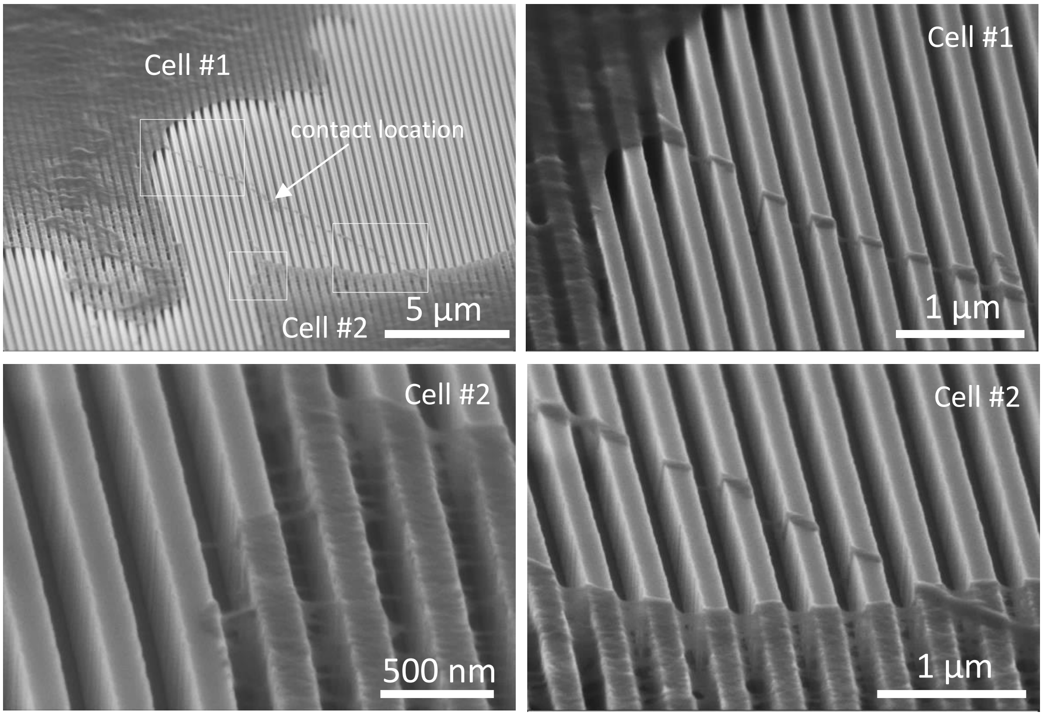

Figure S7. Representative 70° tilted SEM micrograph of a cell incubated for 24 hour on a 0.21 μm comb structure. Note filopodia extended from two different cells contacts on the dense line structure.
